# Effect of *Hottentotta judaicus* Scorpion Venom on Nociceptive Response and Inflammatory Cytokines in Mice Using Experimental Hyperalgesia

**DOI:** 10.3390/molecules30132750

**Published:** 2025-06-26

**Authors:** Lara Haddad, Amira Chender, Rabih Roufayel, Claudine Accary, Adolfo Borges, Jean Marc Sabatier, Ziad Fajloun, Marc Karam

**Affiliations:** 1 Faculty of Health Sciences, University of Balamand, Dekouaneh, Beirut P.O. Box 55251, Lebanon; 2 Faculty of Health Sciences, University of Balamand, Al-Kourah, P.O. Box 100, Tripoli 1300, Lebanon; amirachender@gmail.com (A.C.); claudine.accary@hotmail.com (C.A.); 3 College of Engineering and Technology, American University of the Middle East, Egaila 54200, Kuwait; rabih.roufayel@aum.edu.kw; 4 Faculty of Arts and Sciences, University of Balamand, Al-Kourah, P.O. Box 100, Tripoli 1300, Lebanon; marc.karam@balamand.edu.lb; 5 Laboratorio de Biología Molecular de Toxinas y Receptores, Instituto de Medicina Experimental, Facultad de Medicina, Universidad Central de Venezuela, Caracas 50587, Venezuela; borges.adolfo@gmail.com; 6 Centro para el Desarrollo de la Investigación Científica, Asunción 1255, Paraguay; 7 Inst Neurophysiopathol (INP), CNRS, Aix-Marseille Université, 13385 Marseille, France; 8 Laboratory of Applied Biotechnology (LBA3B), Department of Cell Culture, Azm Center for Research in Biotechnology and Its Applications, EDST, Lebanese University, Tripoli 1300, Lebanon; 9 Department of Biology, Faculty of Sciences 3, Lebanese University, Michel Slayman Tripoli Campus, Ras Maska 1352, Lebanon

**Keywords:** scorpion venom, *Hottentotta judaicus*, inflammatory cytokines, hyperalgesia, LPS, pro-inflammatory, anti-inflammatory, cytokines

## Abstract

Scorpion envenomation is a public health issue in tropical and subtropical regions. Currently, there is limited data on the biological effects of *Hottentotta judaicus* scorpion venom (HjSV) in mammals. This study aims to analyze the effect of HjSV on lipopolysaccharide (LPS)-induced hyperalgesia in mice and its potential modulation of the immunological inflammatory response. Hyperalgesia is characterized by an increased response to pain, accompanied by heightened sensitivity that ranges from mild discomfort to intense pain. A series of tests were conducted, including heat resistance testing in BALB/c mice injected subcutaneously with LPS to induce hyperalgesia and intraperitoneally with HjSV. The hot plate test, used to assess pain endurance in mice, showed that LPS-injected mice, particularly females, exhibited heightened pain sensitivity. This suggests possible sex-based differences in pain perception. When HjSV was administered alone, a reduction in pain sensitivity was observed in both sexes. Additionally, ELISA tests were performed to assess changes in the secretion of inflammatory cytokines IL-4, IL-10, IL-6, IFN-γ, and TNF-α. A consistent increase in both pro- and anti-inflammatory cytokines was observed at early time points in females injected with HjSV alone. Moreover, the hyperalgesia induced by LPS was significantly reduced when HjSV was co-administered, indicating an anti-inflammatory effect at early stages. These findings suggest that HjSV has a significant immunomodulatory effect, potentially exerting anti-inflammatory action during acute inflammation. This effect appears to be time-dependent, diminishing as the immune response transitions toward its adaptive phase.

## 1. Introduction

Scorpion stings are a frequent cause of accidents in subtropical and tropical regions, potentially leading to severe envenomation, particularly in children [1,2]. The severity of symptoms, which can include sweating, nausea, and vomiting, depends on factors such as the scorpion species, venom composition, and victim’s physiological response. Symptoms typically manifest within minutes and can escalate to critical signs, including mydriasis, nystagmus, and respiratory or heart failure, and they can result in death if appropriate pharmacological treatment and/or immunotherapy are not administered promptly [3].

The immune system plays a major role in scorpion envenomation. More specifically, the inflammatory response has been shown to be induced [4,5,6]. In fact, an imbalance between pro- and anti-inflammatory secreted cytokines has been shown to determine the clinical outcome and the progression to severe manifestations, which could lead in certain cases to shock and multiple-organ malfunction [7,8].

Many studies have demonstrated that scorpion envenomation induces the release of pro-inflammatory cytokines such as TNF-α, IL-1, and IL-6, as well as anti-inflammatory cytokines including IL-10. The balance between these cytokines significantly influences the severity of the inflammatory response and associated clinical effects, including cardiac dysfunction and pulmonary edema. In fact, some previous works have reported that TNF-α and IL-1, the primary and earliest secreted mediators, appear to influence organ functions both directly and indirectly during the pathogenesis of scorpion envenomation [3,6], and others showed that after scorpion envenomation, increased secretion of IL-6 has been observed and associated with worsened clinical conditions [4,9]. Meanwhile, IL-6 is often considered an indicator of systemic pro-inflammatory cytokine activation [10], and elevated serum levels of IL-6 have been reported in mice exposed to *Centruroides noxius* and *Tityus serrulatus* scorpion venoms [11,12]. Similarly, increased plasma concentrations of IL-6 have been detected in patients experiencing various severities of envenomation by *Tityus serrulatus* [13,14]. Likewise, elevated levels of IFN-γ—a cytokine that plays a key role in immune defense against infections and tumors—have been also observed as a result of scorpion envenomation [11,12,15].

On an opposite note, certain immune cells are involved in the production of anti-inflammatory cytokines such as IL-1ra, IL-4, IL-10, and TGF-β [5]. These cytokines prevent the exacerbation of the inflammatory response. Increased levels of the regulatory cytokines IL-10 and IL-4 have been observed in murine models injected with the venoms of scorpion such as *Tityus serrulatus*, *Androctonus australis*, and *Centruroides noxius*. IL-10 could also play the role of an immunoregulatory cytokine polarizing the immune response towards the Th2 cytokine response inhibiting IFN-γ [11,16,17,18,19].

Other immunological components have also been implicated in the immune response triggered by scorpion envenomation, contributing to the amplification of inflammation. Notably, activation of the complement system has been shown to play a role by promoting histamine release from mast cells [3,20]. Additionally, innate immune cells such as neutrophils and macrophages are recruited to affected tissues following scorpion envenomation, where their secretion of cytokines further intensifies the inflammatory response [5,21,22].

On the other hand, scorpion venoms are attracting growing interest due to their high content of bioactive peptides capable of interacting with various cellular receptors directly involved in modulating inflammatory signaling pathways [23]. In addition, some toxins found in scorpion venom exert a direct effect on the nociceptive system, notably through their action on voltage-gated ion channels such as sodium and potassium channels, thereby altering the transmission of pain signals [24]. Meanwhile, the diversity of compounds present in scorpion venoms, exhibiting varied spectra of activity in immunomodulation and analgesia, suggests promising therapeutic potential. These animal-derived extracts could therefore serve as a valuable source for the development of novel analgesic strategies specifically targeting the molecular pathways involved in chronic pain and inflammation.

The present study investigates the effect of *Hottentotta judaicus* scorpion venom (HjSV) on the nociceptive response and inflammatory cytokines in mice, using experimental hyperalgesia. *Hottentotta judaicus* is a buthid scorpion species endemic to the Levant region of the Middle East and has previously been characterized for its insecticidal toxins [25]. However, its venom remains poorly studied in terms of its nociceptive and immunological effects. In this work, inflammation was induced by administration of lipopolysaccharide (LPS), resulting in hyperalgesia. Since LPS stimulates the production of various cytokines and eicosanoids by immune cells [26], the set of experiments we carried out examined the possible role of HjSV in LPS-induced hyperalgesia. Although envenomation by *Hottentotta judaicus* is rarely reported in humans, existing case descriptions indicate localized pain, erythema, and paresthesia, with minimal systemic involvement. The limited clinical data reinforces the need for preclinical investigation of HjSV immunological and nociceptive properties.

Hyperalgesia refers to hypersensitivity, a pathology that occurs when pain–sensory nerve pathways are damaged or exposed to mechanical, thermal, or chemical triggers [27]. In most cases of hyperalgesia, patients experience severe pain without any corresponding worsening of the underlying medical condition or injury [28,29].

While various scorpion species such as *Tityus serrulatus*, *Androctonus australis*, and *Centruroides noxius* have been studied for their inflammatory and analgesic profiles, HjSV remains largely unexamined in mammalian models. This lack of data reveals a significant knowledge gap, particularly regarding its potential dual nociceptive and immunomodulatory effects in the context of inflammation. To address this issue, we aim to investigate the in vivo impact of HjSV using a model of LPS-induced hyperalgesia, with a specific focus on pain-related pathophysiological mechanisms and the modulation of pro- and anti-inflammatory cytokine expression. By exploring these uncharted biological effects, this work contributes novel insights into the pharmacological potential of HjSV.

## 2. Results

### 2.1. Establishing the Study Sublethal Dose Through Observed Behavioral Effects

Initially, we aimed to identify sublethal doses of *Hottentotta judaicus* venom that could be used safely in vivo without causing mortality or severe distress. Accordingly, mice were divided into seven groups of ten, each receiving a single, uniform intraperitoneal dose of a specific HjSV concentration. In the first five groups, injected, respectively, with 1 mg/kg, 1.5 mg/kg, 2 mg/kg, 2 mg/kg (for animals provided from a different source), and 2.5 mg/kg, no observable toxicity was noted after 48 h. In the remaining groups, three mice of each gender were injected with higher concentrations: 3.5 mg/kg (group 6) and 7.5 mg/kg (group 7). Group 6 mice exhibited signs of an allergic reaction, as evidenced by ear scratching behavior. Additionally, six hours post injection, one case of paralysis was observed in a female mouse. Beyond the initial signs—paralysis in one female mouse in group 6 and mild allergic reactions—no additional symptoms such as motor impairment, lethality, or abnormal behavior were observed in group 6 or group 7 during the observation period [30]. However, undesirable symptoms were observed in some mice in group 7, which led to the exclusion of the dose used in this group from the rest of the study. Consequently, we chose the dose referred to as HjSV (1), which is equal to 3.5 mg/kg, in our experiments on the basis of the absence of lethality and physiological stress such as mild allergic responses. Our goal was to determine the nonlethal dose suitable for assessing immunological and behavioral effects.

### 2.2. The Effect of HjSV on Pain Sensitivity in Hyperalgesia

**Figure 1:**
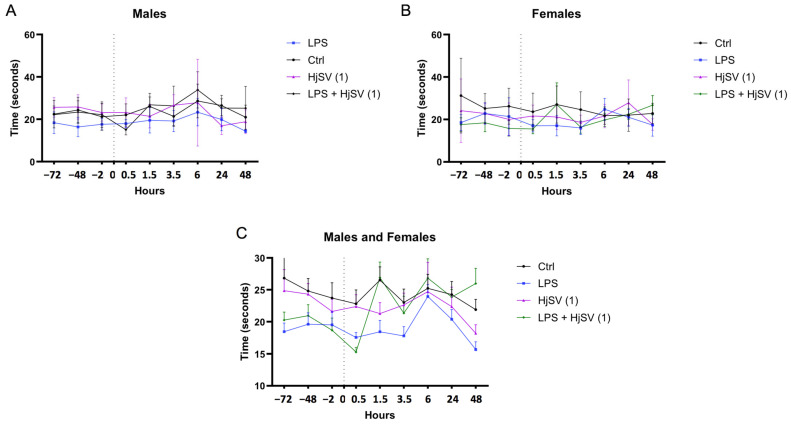
Simplified figure of LPS-induced hyperalgesia in mice models. Changes in pain sensitivity were reported using the hot plate test. Male (**A**) and female (**B**) mice were injected with LPS or/and HjSV at dose 1 = 3.5 mg/kg (HjSV (1)). Panel (**C**) shows the combined data for both male and female mice. After recording baseline sensitivity daily over 3 days prior to any injection, pain sensitivity was tracked up to 2 days post injection. Values are expressed as mean ± SEM; *n* = 5 per gender per group. All mice, including control groups, received subcutaneous injections in the left hind paw—LPS (2.5 mg/mL, 50 µL) for treated groups and phosphate-buffered saline (PBS) for controls—to control for any mechanical or injection-related effects on pain sensitivity. Statistical significance was determined by one-way ANOVA followed by Tukey’s multiple comparison test. Comparisons are made between treatment groups and respective controls unless otherwise specified.

In all groups of mice, the pain threshold was assessed by measuring the time taken for the animal to withdraw its paw from a hot plate. All male mouse groups began at similar baseline levels, with starting latencies ranging from 18 to 26 s across control and treatment groups prior to any injections (Figure 1A). In contrast, greater variability was observed among female groups, such as in the dose of scorpion venom (HjSV (1) = 3.5 mg/kg), which exhibited a baseline response time of 32 s, as shown in Figure 1B. Interestingly, a consistent pattern emerged across all groups and both sexes, characterized by alternating increases and decreases in pain threshold at successive time points. This trend was particularly evident at the 1.5 h, 3.5 h, and 6 h marks, where both male and female mice exhibited an increase followed by a decrease in response times. This pattern was especially pronounced in the group injected with LPS combined with the dose of scorpion venom (HjSV (1)) (Figure 1C), which also induced mild allergic reactions in intraperitoneal sublethality tests, suggesting potential effects on immunoreactivity and cytokine release.

Taking both genders into consideration, LPS-induced hyperalgesia was confirmed by a significant reduction in response latency at 1.5 h and 3.5 h within the same group compared to its baseline. Although the baseline latency of the LPS group appeared lower than that of other groups, this may reflect inter-individual variability rather than pre-existing hyperalgesia.

This finding aligns with the LPS-induced hyperalgesia effects reported by Calil et al. (2014) [31] and Nürnberger et al. (2022) [32]. However, males exhibited less sensitivity to pain at the 1.5 h, 3.5 h, and 6 h time points (Figure 1A). This is consistent with findings suggesting that pain sensitivity may differ between sexes [33], potentially due to the differential expression of INF-γ and IL-17A by CD4 T cells [34].

After LPS injection followed by a dose of HjSV (1) (LPS + HjSV (1)), a sharp increase in the reflex response to heat was recorded at 90 min in both males and females (Figure 1C). This initial response may help explain the noticeable effect of LPS, which triggers the hyperalgesic response. Thermal sensitivity progressively decreased in males, as evidenced by a marked increase in response latency starting at 1.5 h and peaking at 6 h, which suggests a potential anti-inflammatory effect of HjSV.

Although changes in the LPS-only group appear subtle visually, statistical analysis confirmed significant decreases in latency at 1.5 h and 3.5 h compared to the baseline, supporting the presence of LPS-induced hyperalgesia.

Also, we note that variability in baseline nociceptive thresholds was observed between groups, probably due to inter-individual differences. Consequently, hyperalgesia was assessed on the basis of relative changes in latency after injection rather than baseline comparisons alone, in line with established protocols.

### 2.3. Effect of the HjSV on IL-4, IL-10, INF-γ, IL-6, and TNF-α in the Spleens of Different Groups Treated with LPS and/or HjSV (1)

Since pro-inflammatory and anti-inflammatory interleukins have been shown to play a critical role in mediating pain and hyperalgesia, as well as being involved in the immunological alterations induced by scorpion venoms, IL-4, IL-10, INF-γ, IL-6, and TNF-α were quantified by ELISA.

**Figure 2:**
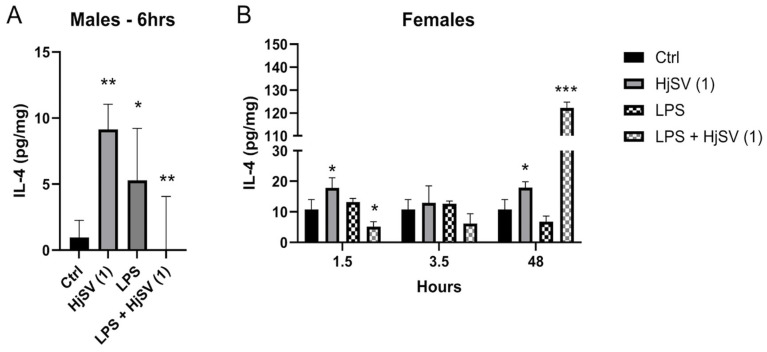
Evaluation of IL-4 level changes in LPS-induced hyperalgesia mouse model. (**A**) IL-4 levels were investigated in male and (**B**) female mice. Values are means ± SEM for n = 5 per group. Statistical significance was determined by one-way ANOVA followed by Tukey’s multiple comparison test. * *p* < 0.05; ** *p* < 0.01; *** *p* < 0.001. Comparisons are made between treatment groups and respective controls unless otherwise specified.

IL-4 is an anti-inflammatory cytokine known to alleviate pain. Upon injection with HjSV (1) alone, secretion of IL-4 showed a significant increase after 6 h (±8 pg/mg) in males (Figure 2A) and after 1.5 h (±19 pg/mg) as well as 48 h (±19 pg/mg) in females (Figure 2B), compared to control mice. Compared to the control, LPS injection also induced an increase in IL-4, although to a lesser extent. When injected with LPS + HjSV (1), IL-4 showed a significant decline after 6 h in males and after 1.5 h in females. Although not significant, a pattern was detected when combined doses of LPS + HjSV (1) were injected in female mice. Even though IL-4 levels dropped significantly at 1.5 h and 3.5 h (approximately ±4 pg/mg compared to the control group), a marked increase was observed after 48 h, reaching around ±120 pg/mg. This suggests the involvement of the adaptive immune response.

**Figure 3:**
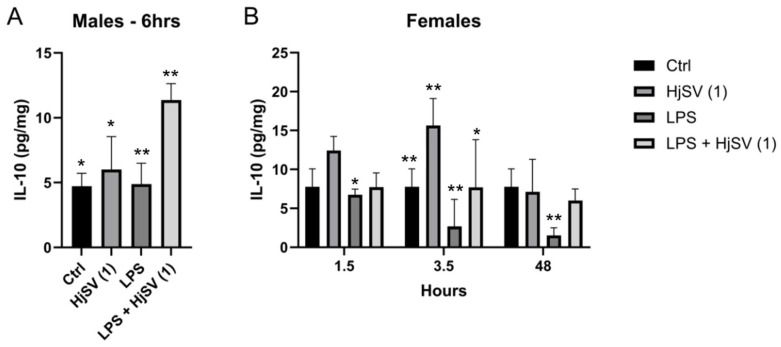
Evaluation of IL-10 level changes in LPS-induced hyperalgesia mice model. (**A**) IL-10 levels were investigated in male and (**B**) female mice. Values are means ± SEM for n = 5 per group. Statistical significance was determined by one-way ANOVA followed by Tukey’s multiple comparison test. * *p* < 0.05; ** *p* < 0.01. Comparisons are made between treatment groups and respective controls unless otherwise specified.

Within this same anti-inflammatory context, a repetitive pattern of IL-10 secretion emerged in both males and females at all time points compared to control mice, i.e., an increase following injection with HjSV (1) alone. This effect became more pronounced at longer time points in females (±12.5 pg/mg and ±15.8 pg/mg after 1.5 h and 3.5 h, respectively) (Figure 3). However, this value decreased drastically to ±7 pg/mg at 48 h. Conversely, a decrease in IL-10 secretion was observed when injected with LPS alone: ±6.5 pg/mg after 1.5 h, ±3 pg/mg after 3.5 h, and ±1.5 pg/mg after 48 h, all of which were less than ±7.8 pg/mg. In all time points and for both genders, a subsequent increase in secretion was recorded when mice were injected with the combination of LPS + HjSV (1). Interestingly, the IL-10 levels, which were low with LPS at 3.5 h and 48 h, were restored when mice were injected with LPS + HjSV (1). Based on this data, we suggest that in the presence of LPS-induced inflammation, HjSV may exert a significant anti-inflammatory effect, especially at later time points (48 h), when the immune response heavily relies on the adaptive immune system.

**Figure 4:**
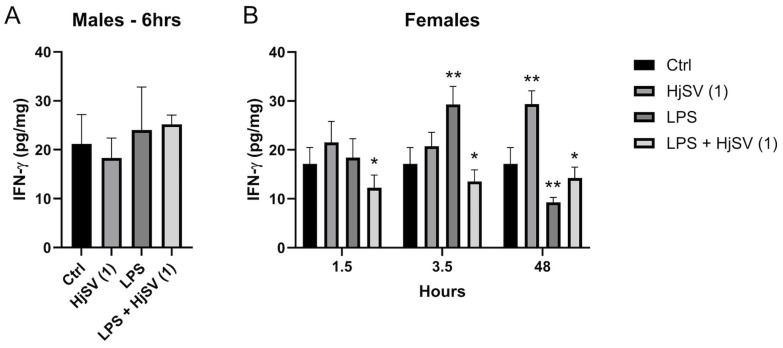
Evaluation of IFN-γ level changes in LPS-induced hyperalgesia mice model. (**A**) IFN-γ levels were investigated in male and (**B**) female mice. Values are means ± SEM for n = 5 per group. Statistical significance was determined by one-way ANOVA followed by Tukey’s multiple comparison test. * *p* < 0.05; ** *p* < 0.01. Comparisons are made between treatment groups and respective controls unless otherwise specified.

IFN-γ is a proinflammatory cytokine known to increase following LPS injection. At early time points, IFN-γ secretion showed an increase, particularly in female mice injected with LPS, such as after 3.5 h, where levels increased from ±17 pg/mg after 1.5 h to ±29.5 pg/mg. This effect was reversed by the addition of HjSV (1) to LPS, with levels decreasing to ±12 pg/mg after 1.5 h and ±14 pg/mg after 3.5 h (Figure 4).

At longer time points, by 48 h, the effect was rectified, with a significant increase in IFN-γ secretion observed with HjSV (1) alone. Conversely, LPS induced a marked decrease in IFN-γ, which was slightly attenuated by the addition of HjSV to LPS (Figure 4).

**Figure 5:**
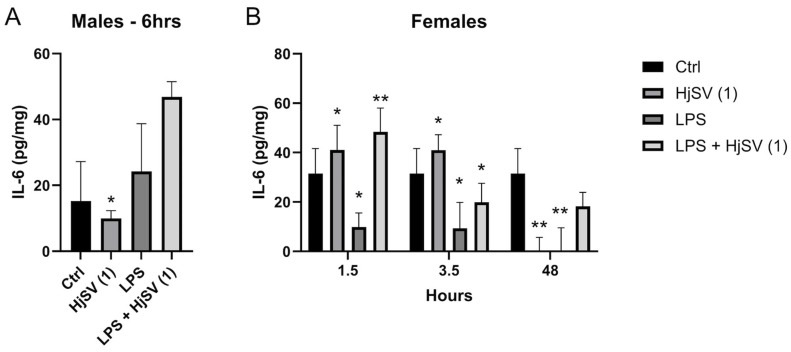
Evaluation of IL-6 level changes in LPS-induced hyperalgesia mice model. (**A**) IL-6 levels were investigated in male and (**B**) female mice. Values are means ± SEM for n = 5 per group. Statistical significance was determined by one-way ANOVA followed by Tukey’s multiple comparison test. * *p* < 0.05; ** *p* < 0.01. Comparisons are made between treatment groups and respective controls unless otherwise specified.

This pattern mirrors the IL-6 secretion patterns observed in females after 1.5 h and 3.5 h. IL-6, a key pro-inflammatory cytokine, showed increased secretion after 1.5 h (±41 pg/mg) and 3.5 h (±40 pg/mg) following HjSV (1) injection alone, but it was completely abolished after 48 h. The LPS-induced group showed similar results to the HjSV group, with approximately the same values at 1.5 h and 3.5 h. However, at later time points, such as 48 h, which are more indicative of the adaptive immune response, IL-6 secretion showed a slight decrease (Figure 5).

Notably, the group injected with the combination of both LPS + HjSV (1) restored IL-6 secretion levels to ±48 pg/mg after 1.5 h, ±22 pg/mg after 3.5 h, and ±20 pg/mg after 48 h, compared to LPS alone. We suggest that, in the presence of LPS-induced inflammation, HjSV may have a significant anti-inflammatory effect, particularly at early time points (3.5 h), which is maintained until 48 h.

As for the male mice (Figure 5A), a contrasting response was observed with HjSV (1). Initially, the concentration was lower than that of the control group of male mice (±8 pg/mg vs. ±15 pg/mg after 3.5 h), but it increased over time, reaching ±10 pg/mg after 6 h and ±30 pg/mg after 48 h. Interestingly, the LPS + HjSV (1) group showed a similar pattern, but with a higher effect than either treatment alone, particularly after 6 h, where it reached ±48 pg/mg compared to ±24 pg/mg in the LPS-only group. This suggests that the inflammatory response in males is sustained up to 6 h, as indicated by the elevated IL-6 levels in the LPS + HjSV (1) group.

TNF-α is known to be a potent pro-inflammatory cytokine. Female mice treated with HjSV (1) alone show significant high levels of secreted TNF-α, ±12 pg/mg, in comparison to the non-treated negative control mice, ±7.2 pg/mg, starting from 1.5 h. Those levels are subject to a decrease after 3.5 h to ±10.5 pg/mg followed by an even sharper decline after 48 h to ±3.9 pg/mg.

**Figure 6:**
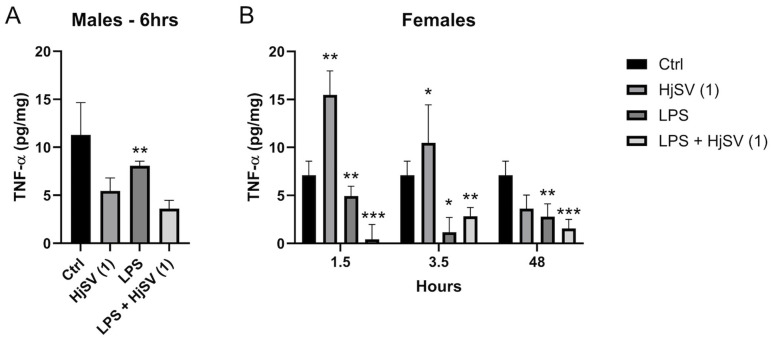
Evaluation of TNF-α level changes upon LPS-induced hyperalgesia mice model. (**A**) TNF-α levels were investigated in male and (**B**) female mice. Values are means ± SEM for n = 5 per group. Statistical significance was determined by one-way ANOVA followed by Tukey’s multiple comparison test. * *p* < 0.05; ** *p* < 0.01; *** *p* < 0.001. Comparisons are made between treatment groups and respective controls unless otherwise specified.

TNF-α induced by LPS is secreted at a lower amount, ±5 pg/mg < ±12 pg/mg, in comparison to HjSV (1) alone at all time points. An interesting pattern was observed, though, when we combined LPS + HjSV (1). In fact, the secretion of TNF-α was almost completely abolished after 1.5 h at ±0.2 pg/mg, increased more than LPS after 3.5 h at ±3 pg/mg > ±1.5 pg/mg, but showed a notable drop after 48 h in comparison to LPS treated female mice alone at ±1.7 pg/mg (Figure 6B).

We could suggest that, in the presence of induced inflammation by LPS, HjSV (1) could have a significant anti-inflammatory effect, especially at early times (1.5 h) where the immune response highly relies on the innate immune system. The same more pronounced effect was observed in male mouse (Figure 6A) treated with LPS + HjSV (1) (±3.7 pg/mg) in comparison with LPS alone after 6 h (±8 pg/mg).

A consistent increase in both pro- and anti-inflammatory cytokines was observed at early time points (1.5 h and 3.5 h) in females when injected with HjSV (1) alone. However, this increase persisted only at longer time points (6 h in males and 48 h in females) for IL-4, IL-10, and IFN-γ, suggesting a shift towards an anti-inflammatory milieu at later stages.

## 3. Discussion

It is well known that inflammatory pain is a common symptom of various inflammatory diseases and can be triggered by different stimuli [31,35,36], including immune-associated stimuli [37]. The short-term increase in sensory sensitivity during inflammation acts as a protective response for the body. However, if inflammation persists, it may lead to sustained hyperalgesia and the onset of chronic pain [38].

LPS is a well-known Toll-like receptor type 4 (TLR4) agonist, used previously as a model to induce inflammation [31], also known as sickness-behavior-associated hyperalgesia [32], by triggering a TLR4/MyD88 (Myeloid Differentiation Primary Response 88)-dependent cytokine cascade in mice. This has been previously shown to induce the release of different cytokines. In our study, we have used LPS as a positive control to induce hyperalgesia in BALB/c mice [31].

Scorpion venoms may be toxic when injected into the body, as they contain low-molecular-mass neurotoxins, bradykinin-potentiating peptides, cytotoxic peptides, and various enzymatic components that collectively contribute to the pathological manifestations of envenomation, including inflammation [39,40,41]. In addition to their toxic effects, scorpion venoms also contain bioactive molecules and peptides with analgesic properties, which are being explored for therapeutic applications. Moreover, scorpion venoms exhibit antibacterial, antiviral, antifungal, and antiparasitic activities, and some contain peptides with anticancer potential, highlighting the broad therapeutic possibilities of scorpion venom-derived components against both established and emerging diseases [40].

In our experiments, subcutaneous LPS injections were performed in the left paw of mice. The injection route was chosen to induce localized inflammatory hyperalgesia, consistent with models of peripheral nociceptive sensitization. Unlike intraperitoneal administration used for systemic inflammation, this approach allows controlled assessment of local pain. Controls received a phosphate-buffered saline (PBS) injection at the same site to control effects related to stinging or mechanical stimulation. In order to explore the potential anti-inflammatory role of HjSV, the same type of mouse was injected intraperitoneally with different doses of scorpion venom, and the sublethal dose of 3.5 mg/kg (HjSV (1)) was chosen in our experiments for the analysis of different splenic cytokines. Behavioral and biochemical evaluations of hyperalgesia in an LPS-induced hyperalgesia BALB/c mice model were then conducted, following an injection of HjSV. BALB/c mice were chosen for their heightened susceptibility to infection and Th2-oriented immune response, enabling accurate detection of cytokine and nociceptive variations after LPS-induced inflammation and exposure to scorpion venom. Our focus is the early immunomodulatory effects of HjSV in this controlled model, without claiming to generalize to all biological systems.

Initially, we confirmed the reliability of our hyperalgesia model by assessing the changes in pain sensitivity in mice using the hot plate test, where heat is the stimulus that activates nociceptors. Notably, we observed a fluctuating pain response pattern that may reflect complex physiological processes, including homeostatic mechanisms. The initial increase in pain sensitivity likely represents an acute inflammatory response. This was followed by a decline, possibly due to a compensatory negative feedback loop aimed at restoring homeostasis, potentially through the release of anti-inflammatory mediators such as IL-4 (as shown in Figure 2, with LPS). A subsequent rise in sensitivity might indicate a secondary wave of inflammation or a delayed effect of the initial insult. It is well established that tissue injury can sustain and amplify pain, and in some cases, prolonged exposure to harmful stimuli can lead to extended activation of primary afferent nociceptors lasting several hours [37,42]. The final decrease may reflect the resolution of inflammation or the action of endogenous analgesic mechanisms, contributing to spontaneous analgesia [43,44,45].

We recognize that baseline variability between groups may influence interpretation. Therefore, hyperalgesia was primarily assessed by within-group changes after injection rather than by between-group comparisons at the baseline. We thus observed that the LPS-treated group, representing the inflammation-induced model, consistently exhibited the highest sensitivity to thermal stimuli. This was evidenced by a significantly shorter latency to respond to the hot plate test at all assessed time points compared to the other experimental groups. This result also indicated that LPS-induced hyperalgesia in the female group was higher than in the males at specific time points (1.5 h, 3.5 h, 6 h), showing that sensitivity to pain might differ between both sexes due to the expression of INF-γ and IL-17A by CD4 T cells [33]. In addition, we saw that the group injected with combined LPS plus HjSV (1), indicated in Figure 4, showed the highest resistance to heat in both females and males, respectively, at 1.5 h and 6 h [46]. These findings suggest the potential anti-inflammatory effect of HjSV. Our results are reminiscent of the previously established anti-inflammatory effect of bee (*Apis mellifera*) venom [47].

It is well known that LPS as well as scorpion venoms are capable of activating different immune cells that migrate and accumulate at the site of injection in the mouse paw and stimulate the RhoA/ROCK signaling pathway in the spinal dorsal horn [36]. This triggers the release of pro-inflammatory cytokines TNF-α and INF-γ. In our study, we were able to observe a significant increase in INF-γ when mice were injected with LPS alone after 3.5 h for females and 6 h for males. This effect is drastically decreased in female mice after 48 h. INF-γ is a pleiotropic cytokine [48]. As demonstrated by Ferrara et al. (2022), [49] INF-γ is an essential regulator for central and peripheral immune response. They have shown that INF-γ plays a critical role in both enhancing the acute neuroinflammatory response and promoting restorative and protective mechanisms during neuropathy healing [49]. Also, several studies have examined gender differences in the immune response to LPS injection and IFN-γ secretion. For example, Von Aulock et al. (2006) observed that *H. sapiens* male blood produced significantly more pro-inflammatory cytokines, such as TNF-α, IL-1β, IL-6, and IL-8, in response to a high concentration of LPS compared to females, while IL-10 and IFN-γ secretion did not differ between the sexes [50]. Another study showed that, after LPS administration, levels of pro-inflammatory cytokines such as IL-1β, IL-6, and TNF-α increased significantly in male and female mice, with sex- and strain-dependent differences [51].

INF-γ production was attenuated at the same timepoints in the same mice injected with LPS + HjSV (1), highlighting the possible anti-inflammatory role of HjSV in acute inflammation. After 48 h, we could observe less prominent release of INF-γ in female mice injected with LPS, slightly reversed with LPS + HjSV (1). This may be attributed to the protective role suggested earlier. This pattern is comparable to that of IL-6 secretion patterns observed in females after 1.5 h and 3.5 h.

IL-6, which is a pro-inflammatory and an anti-inflammatory cytokine induced by different compounds, has an important role during the transition from innate to acquired immunity [46], activated by Th2 cells. It has been shown that IL-6 can strongly inhibit the TGFβ-mediated differentiation of naïve CD4 T cells into regulatory T cells, which inhibit autoimmunity and protect against tissue injury [46]. The induced inflammation by LPS + HjSV (1) had a significant anti-inflammatory effect after 1.5 h.

TNF-α, a pro-inflammatory cytokine, provides further evidence that HjSV may exert an anti-inflammatory effect. This is particularly notable in female mice 1.5 h post injection, when the LPS + HjSV (1) group showed the lowest TNF-α concentration compared to LPS alone. These findings are in line with the study by Wu et al. (2021) [52] which demonstrated that LPS stimulation elevated TNF-α secretion by microglial cells. However, this pro-inflammatory response—along with NF-κB-mediated neuroinflammatory signaling—was inhibited and reversed by a heat-resistant peptide (SVHRP) derived from the scorpion *Olivierus martensii* (formerly *Buthus martensii*) [52]. This indicates the involvement of innate immunity [36], which helps alleviate pain, potentially explaining the increased latency observed on the hot plate obtained in our results and consistent with the findings of Baral et al. (2020) [37]. The reduction in pain by HjSV may be attributed to either the molecular neutralization of cytokines or the suppression of cytokine receptor activation [34,53]. These findings support the hypothesis that HjSV may exert early immunomodulatory effects by modulating both pro- and anti-inflammatory cytokines. The observed temporal variation in cytokine levels suggests that the venom’s action is both dose- and time-dependent, and it could have therapeutic relevance in the context of acute inflammatory responses.

Though TNF-α levels increased following venom administration at early time points, this may reflect an initial immune activation rather than a sustained pro-inflammatory state. Indeed, by 48 h, levels declined significantly. These results suggest a biphasic effect of HjSV, with potential pro-inflammatory activity in the acute phase.

IL-4 is known for its anti-inflammatory properties and neuroprotective role following nerve injury. It is produced by T cells, mast cells, and granulocytes, and it functions by inhibiting the release of pro-inflammatory cytokines. Additionally, IL-4 promotes the differentiation of T cells into the Th2 subtype, which then secrete IL-4 in higher quantities. It also drives macrophage polarization from the pro-inflammatory M1 phenotype to the anti-inflammatory M2 phenotype [54]. Our results showed that mice injected with HjSV (1) had IL-4 upregulated in both sexes at all time points. Combined LPS + HjSV (1) showed a higher concentration of secreted IL-4, especially after 48 h of the female mice. This may clarify the anti-inflammatory effect potentially induced by HjSV, which helps explain the increased pain tolerance.

It is important to highlight that the secretion of cytokines depends also on the toxin composition of scorpion venoms. In fact, a study published by Zoccal et al. (2011) [9] demonstrated that toxins deriving from *Tityus serrulatus* scorpion venom could induce pro- or anti-inflammatory effect depending on the toxin type. They have demonstrated that Ts1 and Ts6 toxins stimulate the release of nitric oxide (NO), IL-6, and TNF-α from macrophage cell line J774.1, potentiated by LPS co-stimulation. In contrast, and upon LPS administration, Ts2, also originating from *T. serrulatus* venom, suppresses the release of NO, IL-6, and TNF-α while upregulating the secretion of IL-10 [9].

Although IL-4 levels were reduced 6 h after injection in male mice, the latter showed elevated pain thresholds, revealing a temporal dissociation between this cytokine and nociceptive behavior. This observation may be explained by the delayed effect of IL-4, involved in long-term immune modulation (Th2 polarization, M2 macrophage activation), as well as by the joint influence of other anti-inflammatory cytokines such as IL-10. In addition, cytokines have been quantified in the spleen, while nociception also results from complex interactions within the central nervous system and peripheral tissues. Our findings indicate that HjSV plays a significant immunomodulatory role, potentially exerting an anti-inflammatory effect during acute inflammation (early timepoints). This effect is time-dependent, becoming less pronounced after 48 h when the immune response shifts towards the adaptive immune system. However, given the complexity of the immune system and the indirect nature of our measurements, these findings should be interpreted with caution and further validated by mechanistic studies.

One proposed mechanism could involve the interaction of HjSV toxins with ion channels expressed on immune cells. For example, it has been extensively demonstrated that the initial event in scorpion envenomation involves the targeting of ion channels by specific neurotoxins. These neurotoxins can influence the immune system through their interaction with voltage-gated sodium channels on neuronal terminals, potentially causing axonal membrane depolarization and triggering the release of neuropeptides and neurotransmitters. These released agents have been shown to stimulate the production of immune mediators, including cytokines. Particularly, voltage-gated potassium channels are known to be involved in regulating immune function. In vitro studies have shown that blocking these channels reduces T cell activation and delays hypersensitivity responses [55]. In this context, Butantoxin, found in the venoms of three Brazilian scorpions (*T. serrulatus*, *T. bahiensis*, and *T. stigmurus*), has been demonstrated to reversibly inhibit potassium channels, suppress T cell proliferation, and decrease IL-2 production [56].

While limited by a relatively small sample size and biological variability—particularly among female mice—and constrained by gender-based comparisons due to differing timepoints, this study’s strength lies in its integrative behavioral and immunological analysis using a poorly characterized venom in a validated inflammatory model. Further research is needed to identify the specific HjSV components responsible for the observed anti-inflammatory effects, and additional studies are also warranted to test its impact in chronic pain or autoimmune inflammation models, as well as to explore its interaction with nociceptive ion channels using electrophysiological or molecular techniques. One limitation of this study is the non-parallel sampling of males and females across all time points. While males were included at a representative 6 h time point, future studies will adopt a fully sex-balanced time course design.

## 4. Materials and Methods

### 4.1. HjSV Preparation

The venom used was provided by the Lebanese Venom Company (LVC) (Karaoun, Lebanon) for exclusive research use and is not registered under a Drug Master File with the Lebanon FDA. Nonetheless, batch-to-batch consistency was ensured through standardized collection, lyophilization, and aliquoting procedures under sterile conditions, and each batch was stored at −20 °C until use. Venom batches were dissolved in phosphate saline buffer prior to their use. For further accuracy, each batch was aliquoted into several tubes, each one containing 1 mL to avoid freezing and thawing of samples.

### 4.2. Mice and Their Handling

All animals were handled, and experimental procedures were carried out according to the guidelines of the Institutional Animal Care and Use Committee at the University of Balamand (UOB), with strict adherence to the ethical guidelines for the study of experimental pain in conscious animals.

BALB/c mice are frequently used in immunological and infectious disease models due to their high sensitivity to inflammation and their well-characterized immune response. The mice used in this study were bred in the animal facility of the UOB [57].

### 4.3. Sublethal Dose Determination

To select appropriate venom dose for in vivo experiments, a preliminary study was conducted on BALB/c mice (n = 10 per group) using seven different concentrations of HjSV administered via the intraperitoneal route. Mice were observed over 48 h for behavioral changes, signs of distress, allergic reactions, and mortality. No deaths were recorded at any tested dose. Mild allergic signs (e.g., ear scratching, temporary paralysis) were observed at 3.5 mg/kg. Based on these findings, the sublethal dose of 3.5 mg/kg was selected for subsequent experiments.

### 4.4. Hot Plate Test

For this test, mice were divided into six groups. Each group contained 10 mice (five males and five females with each gender in a separate cage), and the hot plate test was performed for each group six times after injecting the mice with LPS and venom separately or mixed together at different time points (30 min, 1.5 h, 3.5 h, 6 h, 24 h, and 48 h). Each measurement consisted of placing the mouse on the hot plate and recording the latency time to a nociceptive response (paw licking or jumping), with a cutoff at 40 s to avoid injury.

### 4.5. Injection of a New Batch of Mice for Dissection

A total of 65 mice (20 males, 45 females) were divided into three experimental groups, each subdivided into three subgroups according to sacrifice times. A control group (n = 8; 3 males, 5 females) was also included. Group 1 (n = 8) served as a negative control with no treatment. Group 2 (n = 18; 15 females, 3 males) received a subcutaneous injection of LPS (2.5 mg/mL, 50 µL/mouse). No visible inflammatory signs were observed. Females were sacrificed after 1.5 h, 3.5 h, and 48 h (5 mice/time), and males were sacrificed after 6 h (3 mice). Group 3 (n = 20; 15 females, 5 males) received an intraperitoneal injection of diluted venom (3.5 mg/kg, 2 mL). Females were sacrificed after 1.5 h, 3.5 h, and 48 h (5 mice/time), and males were sacrificed after 6 h. Group 4 (n = 19; 15 females, 4 males) received both LPS (50 µL subcutaneously) and venom (2 mL intraperitoneally). Females were sacrificed at the three indicated times (10 mice in total), and males were sacrificed after 6 h. These 65 mice were used for immunological and behavioral testing.

Spleens of all the sacrificed mice were extracted using the same procedure in order to detect the secretion of several cytokines.

To model localized inflammation, LPS was injected subcutaneously in the left hind paw (50 µL of 2.5 mg/mL). Control animals received the same volume of PBS by the same route, to control for potential effects of injection alone.

Due to preliminary findings suggesting stronger cytokine responses in female mice, we focused on three time points (1.5 h, 3.5 h, and 48 h) for females and a single representative time point (6 h) for males. This approach was adopted to maximize experimental resolution under ethical and logistical constraints.

### 4.6. Dissection and Organ Extraction

Mice were anesthetized using chloroform vapor until complete unconsciousness, followed by sacrifice through cardiac puncture. This method was approved by the Institutional Animal Care and Use Committee of the UOB and complies with national animal research guidelines.

### 4.7. Homogenization of Organs

Collected spleens were individually homogenized using T-10 basic ULTRA-TURRAX (Janke & Kunkel-Str. 10, 79219 Staufen, Germany) for 1 min at 20,000 rpm in 1.2 mL of homogenization buffer, which consisted of 0.2% of NaCl, 0.05% bovine serum albumin (BSA), and 0.05% Tween 20, supplied with protease inhibitor in PBS. The homogenized samples were centrifuged at 14,500 rpm for 15 min at 4 °C; supernatants were removed and stored in pyrogen/endotoxin-free tubes at −20 °C until further usage.

### 4.8. Enzyme-Linked Immunosorbent Assay (ELISA)

To detect the cytokines, IL-4, IL-10, IFN-γ, IL-6, and TNF-α, direct ELISAs were performed using Pepro-tech kits. The total amount of proteins presents in each organ was quantified using the Bradford assay. Each kit contained four tubes: capture antibody (Ab), standard Ab, detection Ab, and avidin. In addition to these kits, we had a buffer kit containing the blocking (1×), washing solution (20×), Diluent (20×), PBS (20×), substrate (1×), and 10 plates. ELISA steps were performed according to the manufacturer instructions. Briefly, the capture antibody was loaded in the plate and incubated for overnight. Ninety-six-well plates were washed, and blocking solution was added for 1 h. Then, the plates were washed again, and standards and samples were added and incubated for 2 h. After multiple washes and the addition of the enzyme and its substrate, plates were read on a spectrophotometer every 5 min for 30 min.

### 4.9. Statistical Analysis

All statistical analyses were performed using GraphPad Prism 6.00 software (GraphPad Software Inc., San Diego, CA, USA). For comparisons involving more than two groups, one-way ANOVA was applied, followed by Tukey’s post hoc multiple comparisons test to assess pairwise group differences. Results are presented as mean ± SEM. A *p*-value < 0.05 was considered statistically significant.

## 5. Conclusions

In this study, *Hottentotta judaicus* scorpion venom (HjSV) demonstrated potential anti-inflammatory effects in an LPS-induced hyperalgesia mouse model, particularly through the modulation of IL-4, IL-10, and TNF-α levels. While some cytokine trends and behavioral outcomes suggest time- and dose-dependent immunomodulatory effects, these findings must be interpreted cautiously due to biological variability and limited sample size. Future investigations will focus on isolating the specific bioactive peptides responsible for these effects and on evaluating their therapeutic relevance in chronic pain and systemic inflammatory models using more robust experimental designs.

## Data Availability

The data are not publicly available due to ethical restrictions but may be obtained from the corresponding author upon reasonable request and subject to institutional approval.

## Abbreviations

The following abbreviations are used in this manuscript:

HjSV	*Hottentotta judaicus* scorpion venom
LPS	Lipopolysaccharide
ELISA	Enzyme-Linked Immunosorbent Assay
IL-4	Interleukin 4
IL-10	Interleukin 10
IL-6	Interleukin 6
IFN-γ	Interferon gamma
TNF-α	Tumor necrosis factor alpha
IL-17A	Interleukin 17A
TLR4	Toll-like receptor type 4
MyD88	Myeloid differentiation primary response 88
PBS	Phosphate-buffered saline
RhoA	Ras homolog gene family member A
ROCK	Rho-associated coiled-coil-containing protein kinase
NO	Nitric oxide
