# Correction: Haddad et al. Effect of *Hottentotta judaicus* Scorpion Venom on Nociceptive Response and Inflammatory Cytokines in Mice Using Experimental Hyperalgesia. *Molecules* 2025, *30*, 2750

**DOI:** 10.3390/molecules30193874

**Published:** 2025-09-25

**Authors:** Lara Haddad, Amira Chender, Rabih Roufayel, Claudine Accary, Adolfo Borges, Jean Marc Sabatier, Ziad Fajloun, Marc Karam

**Affiliations:** 1 Faculty of Health Sciences, University of Balamand, Dekouaneh, Beirut P.O. Box 55251, Lebanon; 2 Faculty of Health Sciences, University of Balamand, Al-Kourah, P.O. Box 100, Tripoli 1300, Lebanon; amirachender@gmail.com (A.C.); claudine.accary@hotmail.com (C.A.); 3 College of Engineering and Technology, American University of the Middle East, Egaila 54200, Kuwait; rabih.roufayel@aum.edu.kw; 4 Faculty of Arts and Sciences, University of Balamand, Al-Kourah, P.O. Box 100, Tripoli 1300, Lebanon; marc.karam@balamand.edu.lb; 5 Laboratorio de Biología Molecular de Toxinas y Receptores, Instituto de Medicina Experimental, Facultad de Medicina, Universidad Central de Venezuela, Caracas 50587, Venezuela; borges.adolfo@gmail.com; 6 Centro para el Desarrollo de la Investigación Científica, Asunción 1255, Paraguay; 7 Inst Neurophysiopathol (INP), CNRS, Aix-Marseille Université, 13385 Marseille, France; 8 Laboratory of Applied Biotechnology (LBA3B), Department of Cell Culture, Azm Center for Research in Biotechnology and Its Applications, EDST, Lebanese University, Tripoli 1300, Lebanon; 9 Department of Biology, Faculty of Sciences 3, Lebanese University, Michel Slayman Tripoli Campus, Ras Maska 1352, Lebanon

In the original publication [1], there was an error in the Institutional Review Board Statement. Before this work was carried out, the proposal was submitted to the Proposal Faculty Research Graduate Committee for evaluation. This committee evaluates all proposed research, taking into consideration both the ethical aspects of the work and the specific guidelines established for studies conducted in the university’s animal facility. Following this assessment, the project received clearance and was authorized to proceed (Protocol code: 2023/10; date of approval: December 2023). Upon the establishment of the Institutional Animal Care and Use Committee (IACUC) at the University of Balamand, the project was retrospectively reviewed based on available documentation, procedures, and ethical considerations. The Committee confirmed that the animal use practices and procedures adhered to generally accepted standards of animal care and use. The approval was then granted (Protocol code: IACUC07/2025; date of approval: 1 September 2025).

The correct Institutional Review Board Statement appears below: Before this work was carried out, the proposal was submitted to the Proposal Faculty Research Graduate Committee for evaluation. This committee evaluates all proposed research, taking into consideration both the ethical aspects of the work and the specific guidelines established for studies conducted in the university’s animal facility. Following this assessment, the project received clearance and was authorized to proceed (Protocol code: 2023/10; date of approval: December 2023). Upon the establishment of the Institutional Animal Care and Use Committee (IACUC) at the University of Balamand, the project was retrospectively reviewed based on available documentation, procedures, and ethical considerations. The Committee confirmed that the animal use practices and procedures adhered to generally accepted standards of animal care and use. The approval was then granted (Protocol code: IACUC07/2025; date of approval: 1 September 2025).

The authors state that the scientific conclusions are unaffected. This correction was approved by the Academic Editor. The original publication has also been updated.

## References

[1] Haddad L., Chender A., Roufayel R., Accary C., Borges A., Sabatier J.M., Fajloun Z., Karam M. (2025). Effect of *Hottentotta judaicus* Scorpion Venom on Nociceptive Response and Inflammatory Cytokines in Mice Using Experimental Hyperalgesia. Molecules.

